# *Arachniodes × tohtomiensis* hyb. nov. (Dryopteridaceae) from Japan

**DOI:** 10.3897/phytokeys.269.176789

**Published:** 2026-01-16

**Authors:** Kiyotaka Hori, Daiki Takahashi, Ying-Hong He, Thant Shin, Yoshihisa Suyama

**Affiliations:** 1 The Kochi Prefectural Makino Botanical Garden, 4200–6 Godaisan, Kochi, Kochi 781–8125, Japan The Kochi Prefectural Makino Botanical Garden Kochi Japan; 2 Faculty of Agriculture, Kyushu University, West Zone 5–827, 744, Motooka, Nishi-ku, Fukuoka, 819–0395, Japan Kyushu University Fukuoka Japan; 3 Kawatabi Field Science Centre, Graduated School of Agricultural Science, Tohoku University, 232–3, Yomogida, Narukoonsen, Osaki, Miyagi, 989–6711, Japan Tohoku University Miyagi Japan; 4 Forest Research Institute, Yezin, Nay Pyi Taw, Myanmar Forest Research Institute Yezin Myanmar

**Keywords:** Diploid, neighbor-net analysis, new hybrid, MIG-seq, STRUCTURE analysis

## Abstract

This study aims to characterize a hybrid of *Arachniodes* (Dryopteridaceae), *A. × tohtomiensis*, for which molecular evidence of its origin and detailed morphological descriptions are lacking. The genome composition of *A. × tohtomiensis*, clarified based on plastid DNA and MIG-seq analyses, indicates that it is a diploid hybrid of *A. exilis* and an undetected species. This origin was also supported by phylogenetic, STRUCTURE, principal component, and neighbor-net analyses. Based on these evidences, it is formally described as *A. × tohtomiensis* Shimura & Hori, **hyb. nov**. It is morphologically characterized by its yellowish-green laminae and erose-to-ciliate indusia.

## Introduction

The fern genus *Arachniodes* (Dryopteridaceae) includes several species that are taxonomically challenging to differentiate, particularly in East and Southeast Asia. Identifying species in *Arachniodes* based solely on morphology is difficult because of their wide morphological variation, the absence of clear diagnostic traits, and the frequent occurrence of polyploidization and hybridization.

The 19 putative hybrids of *Arachniodes* listed in Japan help resolve the morphological overlap among species, although most lack DNA-based evidence (Ebihara 2017; Ebihara and Nitta 2019; Ebihara et al. 2023). However, MIG-seq analyses showed that several hybrid and allopolyploid species occur in Japan (Hori 2024; Hori et al. 2025). Among the 19 hybrids, *A. × tohtomiensis* nom. nud. is considered a diploid hybrid of *A. exilis* and an unidentified species, but molecular evidence supporting this hypothesis has not been provided (Shimura et al. 1982; Tsutsui 1988; Nakaike 1992; Ebihara 2017). Furthermore, morphological descriptions of diagnostic traits are also lacking (Shimura et al. 1982; Tsutsui 1988; Nakaike 1992; Ebihara 2017; Ebihara and Nitta 2019; Ebihara et al. 2023). This hybrid was firstly reported as ‘*Arachniodes* ×sp.’ from Japan by Shimura et al. (1982), who named it ‘enshu-kanawarabi’ in Japanese. Their cytological observations showed irregular meiosis, suggesting a diploid hybrid origin. In addition, Shimura et al. (1982) reported *A. tsutsuiana* nom. nud., as a diploid sexual species with intermediate morphological features between *A. aristata* (G. Forst.) Tindale and *A. sporadosora* (Kunze) Nakaike. Subsequently, Tsutsui (1988) mentioned this undescribed hybrid as *A. × tohtomiensis* Shimura nom. nud. Tsutsui (1988) morphologically compared *A. × tohtomiensis* with *A. aristata*, *A. sporadosora*, and *A. tsutsuiana* nom. nud., concluding that *A. × tohtomiensis* can be distinguished by its ciliate indusia from *A. exilis*, *A. sporadosora*, and *A. tsutsuiana*, whereas *A. tsutsuiana* is a hybrid of *A. aristata* and *A. sporadosora*. However, Tsutsui (1988) did not provide a Latin descriptions and designate holotypes for the two undescribed hybrids.

More recently, Serizawa (2009) revised the taxonomy of *Arachniodes aristata*, excluding it from the Japanese flora and applying the name *A. exilis* (Hance) Ching to the Japanese species previously called *A. aristata*. He also treated *A. oohorae* H. Itô as a form of *A. exilis*: *A. exilis* (Hance) Ching f. *oohorae* (H. Itô) Seriz. Ebihara (2017) subsequently considered (1) *A. exilis* f. *oohorae* synonymous with *A. exilis*, (2) *A. tsutsuiana* as *A. exilis* × *A. sporadosora*, and (3) *A. × tohtomiensis* as hybrids of *A. exilis* and an unidentified species. Chang et al. (2021) treated *A. exilis* and *A. sporadosora* as synonyms of *A. carvifolia* (Kunze) Ching and *A. cornu-cervi* (D. Don) Fraser-Jenk., respectively, although without molecular evidence. In addition, diploid sexual cytotypes of *A. aristata* and *A. sporadosora* have been reported from Japan (Mitui 1965; 1970; Kurita 1966; Hirabayashi 1970).

The aim in this study was to clarify the taxonomic identity of *Arachniodes × tohtomiensis* and its parental relationships. This study provides molecular evidence based on plastid DNA and MIG-seq analyses as well as detailed morphological descriptions with the designation of a holotype of *A. × tohtomiensis*.

## Materials and methods

Leaf material was collected from *Arachniodes cornu-cervi*, *A. exilis* including *A. oohorae*, *A. sporadosora*, *A. exilis* × *A. sporadosora* (i.e. *A. tsutsuiana*), and *A. × tohtomiensis*. Voucher specimens were deposited with the Kochi Prefectural Makino Botanical Garden (MBK).

The nuclear DNA content of *Arachniodes exilis*, *A. sporadosora*, *A. exilis* × *A. sporadosora*, and *A. × tohtomiensis* was estimated using flow cytometry, as described by Yamamoto et al. (2024) for several samples. Approximately 400 mm^2^ of fresh leaf tissue was chopped with a razor blade in 1 mL of Triton X-100 buffer together with leaf tissue of *Vicia faba* Inovec (2C = 26.9 pg; Doležel et al. 1992) as an internal standard. The filtrates were stained with propidium iodide, and approximately 3,000 nuclei were analyzed three times per sample using a Sysmex CyFlow Ploidy Analyzer (Sysmex, Japan). Additionally, the shapes of sporangia and spores of *A. × tohtomiensis* specimens were examined.

The total DNA was extracted from the silica-dried leaves using 2× CTAB buffer, following the methods of Doyle and Doyle (1990). The plastid *rbcL* gene and *trnL–trnL-F* intergenic spacer were amplified and sequenced as maternally inherited cpDNA markers (Gastony and Yatskievych 1992; Hori et al. 2018; Lu et al. 2019). The phylogenetic analyses were conducted using reference accessions from Lu et al. (2019). The sequences were aligned using MUSCLE (Edgar 2004) and analyzed using maximum-likelihood (ML) and Bayesian inference (BI) methods. The ML and BI analyses were conducted with MEGA 12 (Kumar et al. 2024) and MrBayes 3.2.7 (Ronquist et al. 2012), respectively. The best-fit nucleotide substitution models (K80 + G + I for *rbcL*, HKY + G + I for *trnL–trnL-F*) were determined using jModelTest 2.1.10 (Darriba et al. 2012) based on the BIC values and applied in BI analyses. Because these models were unavailable in MEGA, the Tamura 3-parameter + G + I model was used for the ML analyses. Four Markov chains were simultaneously run for 1 million generations, sampling every 100 generations. Tracer 1.7.1 (Rambaut et al. 2018) was used to examine the posterior distributions and effective sample sizes, with the first 2,500 trees discarded as burn-in. The node support in the ML analyses was evaluated using 105 adaptive bootstrap replicates (threshold = 5.00). *Rumohra adiantiformis* (first stage) Ching was used as the outgroup according to Lu et al. (2019).

Genome-wide single-nucleotide polymorphism (SNP) data were obtained using MIG-seq to infer the genomic composition of *Arachniodes × tohtomiensis* (Suyama and Matsuki 2015; Suyama et al. 2022). Twenty-seven samples covering *A. exilis*, *A. sporadosora*, *A. exilis* × *A. sporadosora*, and *A. × tohtomiensis* were analyzed for population structure following the methods of Hori et al. (2025).

Low-quality and short reads were removed using Trimmomatic 0.39 (Bolger et al. 2014) using the same parameters as Hori et al. (2025). Trimmed paired-end reads were concatenated using CAT commands. Adapter trimming, quality filtering, de novo assembly, genotyping, and STRUCTURE input preparation were conducted with ipyrad v. 0.9.105 (Eaton and Overcast 2020) using the following key parameters: datatype = ddrad; filter for adapters/primers = 1; minimum depth for majority-rule base calls = 3; clust_threshold = 0.9; minimum read length after adapter trimming = 60. Branches were analyzed for each species in steps 4 and 5 and merged in step 6. The parameter min_samples_locus was set to 1 and 4 for steps 4–5 and 6–7, respectively; and all other parameters were set to the default.

STRUCTURE v. 2.3.4 (Pritchard et al. 2000) and principal component analysis (PCA) were plotted using the Jupyter notebook toolkit implemented in ipyrad. The hdf5 file was exported in step 7. The minimum coverage parameter (mincov) was tested from 0.7 to 0.96, and 0.9 was adopted, before STRUCTURE analyses. Unlinked SNPs were subsampled using the parameters described by Hori et al. (2025). The optimal K value was determined using the delta K method, and the mean log-likelihood probabilities were calculated using ipyrad. The PCA plots were generated with the following parameters: mincov = 0.6, impute_method = “sample,” and nreplicates = 100.

The .vcf file exported from ipyrad was converted to PHYLIP format and then to NEXUS format using PGDSpider 2.1.1.5 (Lischer and Excoffier 2012). Neighbor-net networks (Bryant and Moulton 2004) were constructed in SplitsTree4 v. 4.19.2 (Huson and Bryant 2006) using the uncorrected P-distance matrices calculated from the concatenated SNP sequences including invariant sites. Ambiguous sites were treated as missing data.

Finally, the apex of the laminae, color of the laminae, dissection of the laminae, most basiscopic pinnules, margin of indusia, stipe spacing on the rhizome, and rhizome morphology were compared among *Arachniodes exilis*, *A. sporadosora*, *A. exilis* × *A. sporadosora*, and *A. × tohtomiensis* nom. nud.

## Results

**Figure 1.**
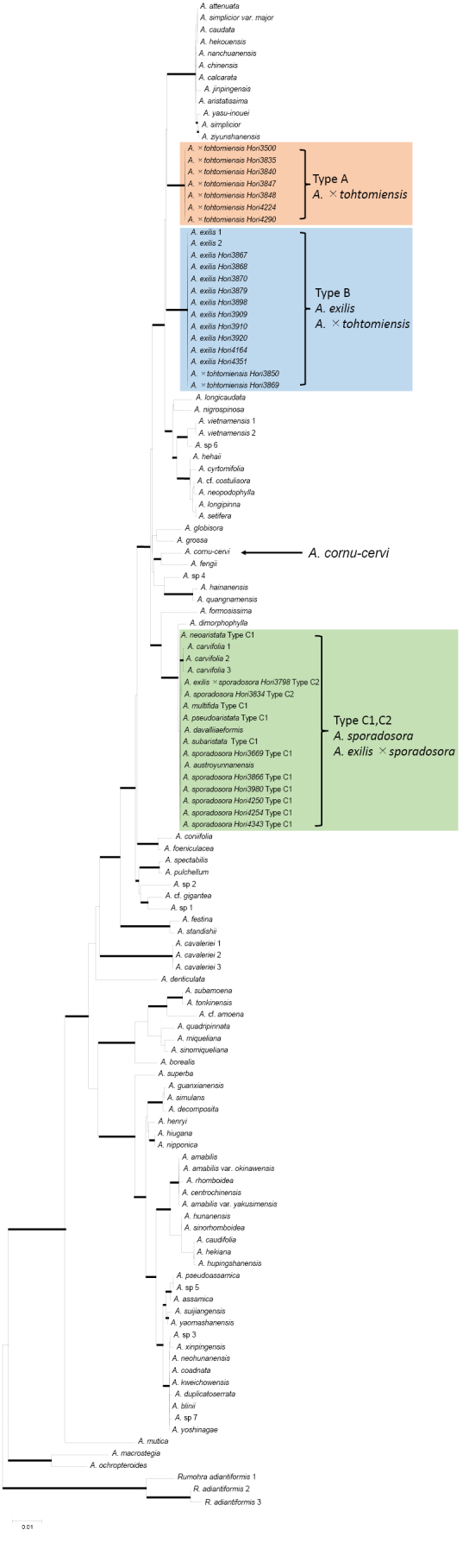
The ML tree (log likelihood = −8487.91) based on the sequences of the plastid DNA. Thick lines indicate strong support (MLBS≥85% and BIPP≥95%). Haplotype **A–C**. are placed in strongly supported different clades, respectively.

The mean DNA contents of *Arachniodes exilis*, *A. sporadosora*, *A. exilis* × *A. sporadosora*, and *A. × tohtomiensis* were 16.1 pg (n = 3), 17.5 pg (n = 4), 16.5 pg (n = 1), and 15.9 pg (n = 3), respectively. The plastid DNA alignment was 2,005 bp long, of which 380 bp (18.9%) were parsimony-informative. An ML tree with bootstrap percentages (MLBS) and Bayesian posterior probabilities (BIPP) is shown in Fig. 1. Three plastid haplotype types were identified: A (*A. × tohtomiensis*), B (*A. exilis* or *A. × tohtomiensis*), and C (*A. exilis* × *A. sporadosora* and *A. sporadosora*). One haplotype (C1) of *A. sporadosora* is shared by *A. multifida* Ching, *A. neoaristata* Ching, *A. pseudoaristata* (Tagawa) Ohwi, *A. subaristata* Ching, and Y. T. Hsieh. *A. carvifolia* is closely related to *A. sporadosora* but their haplotypes are distinct (Fig. 1). *A. cornu-cervi* was placed at a separate position from A, B, and C.

The raw MIG-seq data were processed using Ipyrad. The total reads, clusters, read consensus sequences, and loci in the assembly were 33,457,768, 2,456,008, 652,046, and 337,985, respectively. The average number of reads per sample was 1,239,177 with 90,963 clusters, 24,149 read consensus sequences, and 12,517 loci. The SNP matrix contained 177,473 sites (69.6% missing), and the sequence matrix contained 3,216,355 sites (71.2% missing). The raw data were deposited in the DDBJ Sequence Read Archive (BioProject accession number: PRJDB20422).

**Figure 2.**
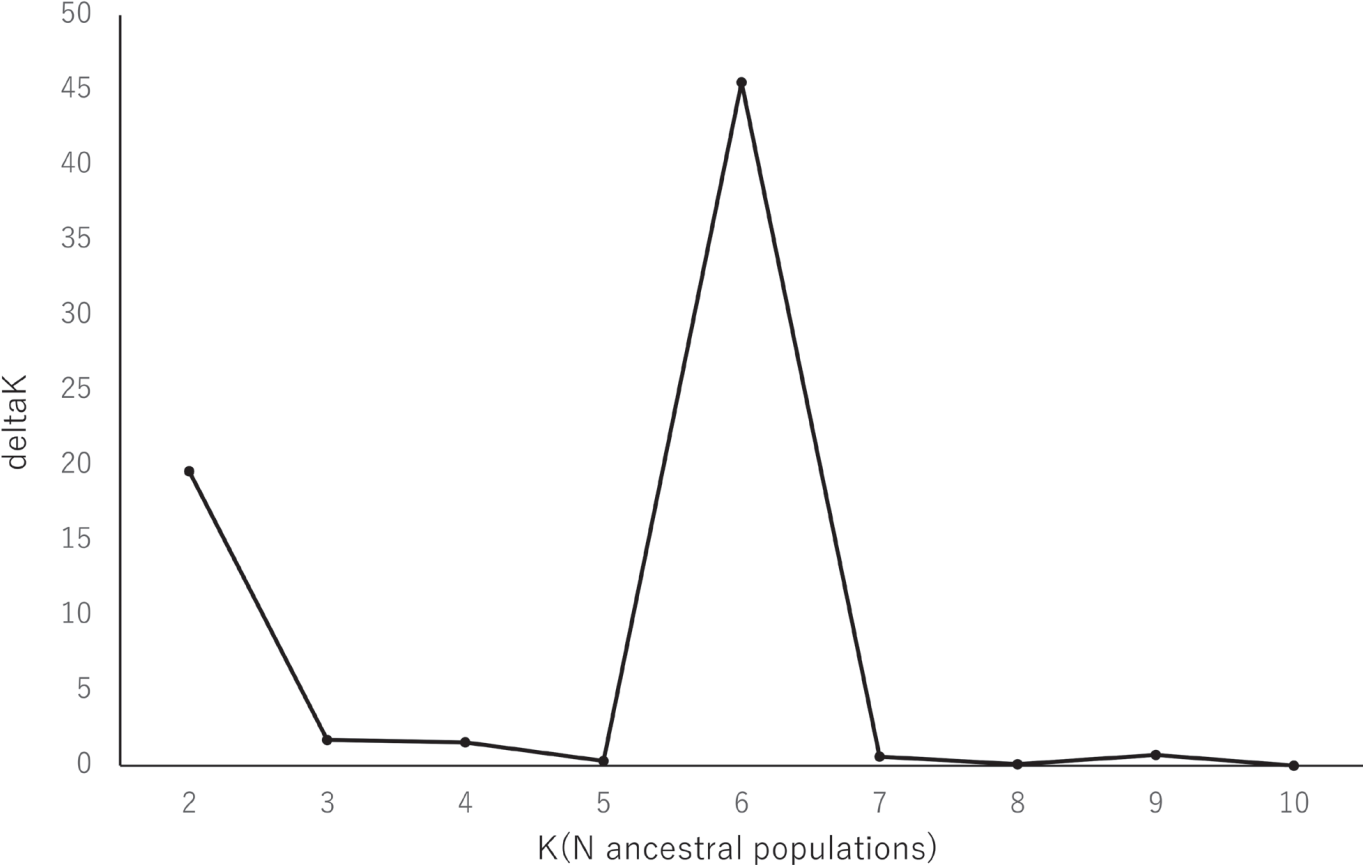
Delta K for each K in STRUCTURE analyses of *Arachniodes × tohtomiensis* and its relatives using SNP data from MIG-seq.

**Figure 3.**
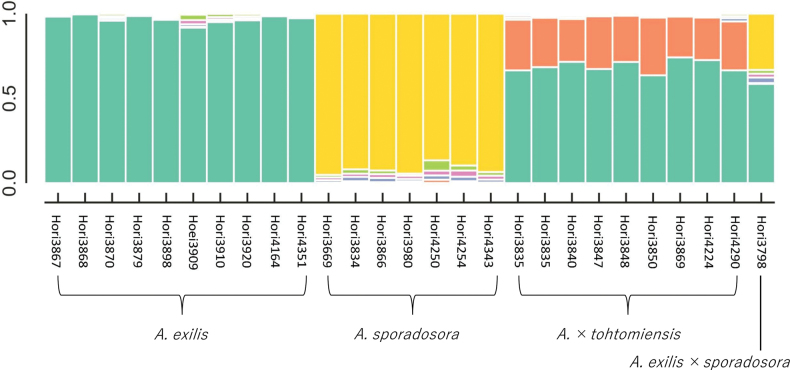
STRUCTURE analyses of *Arachniodes × tohtomiensis* and its relatives using SNP data from MIG-seq.

Bayesian clustering analysis of 314 unlinked SNPs after filtering indicated that K = 6 was the optimal number of clusters (Evanno et al. 2005; Fig. 2). Of the three major clusters (green, yellow, orange), *Arachniodes exilis* and *A. sporadosora* were assigned to the green and yellow clusters, respectively. Their hybrid (*A. exilis* × *A. sporadosora*) exhibited a mixture of the characteristics of these clusters, whereas *A. × tohtomiensis* was associated with green and orange clusters (Fig. 3). No individuals were found in the orange cluster. The other three clusters were small and not specific for each species and hybrid.

**Figure 4.**
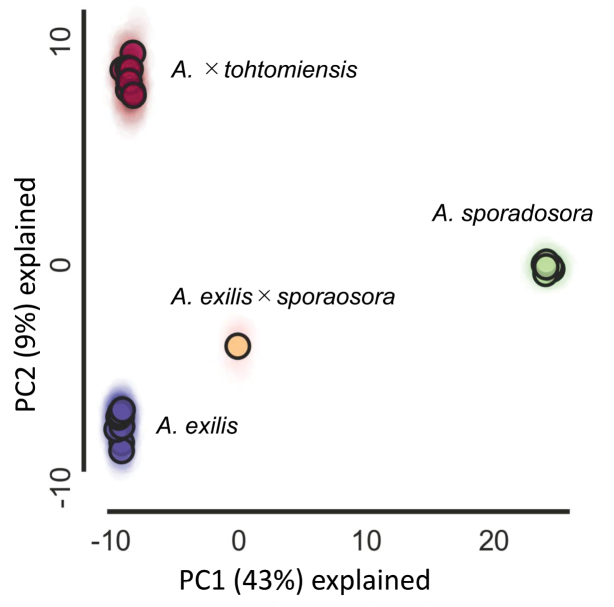
PCA plots of *Arachniodes × tohtomiensis* and its relatives using SNP data from MIG-seq with PC1 and PC2.

PCA based on 1,746 unlinked SNPs showed that each taxon occupied a distinct position (Fig. 4). *Arachniodes exilis* × *A. sporadosora* was intermediate between the parental species in both PC1 and PC2, whereas *A. × tohtomiensis* clustered closer to *A. exilis* in PC1.

**Figure 5.**
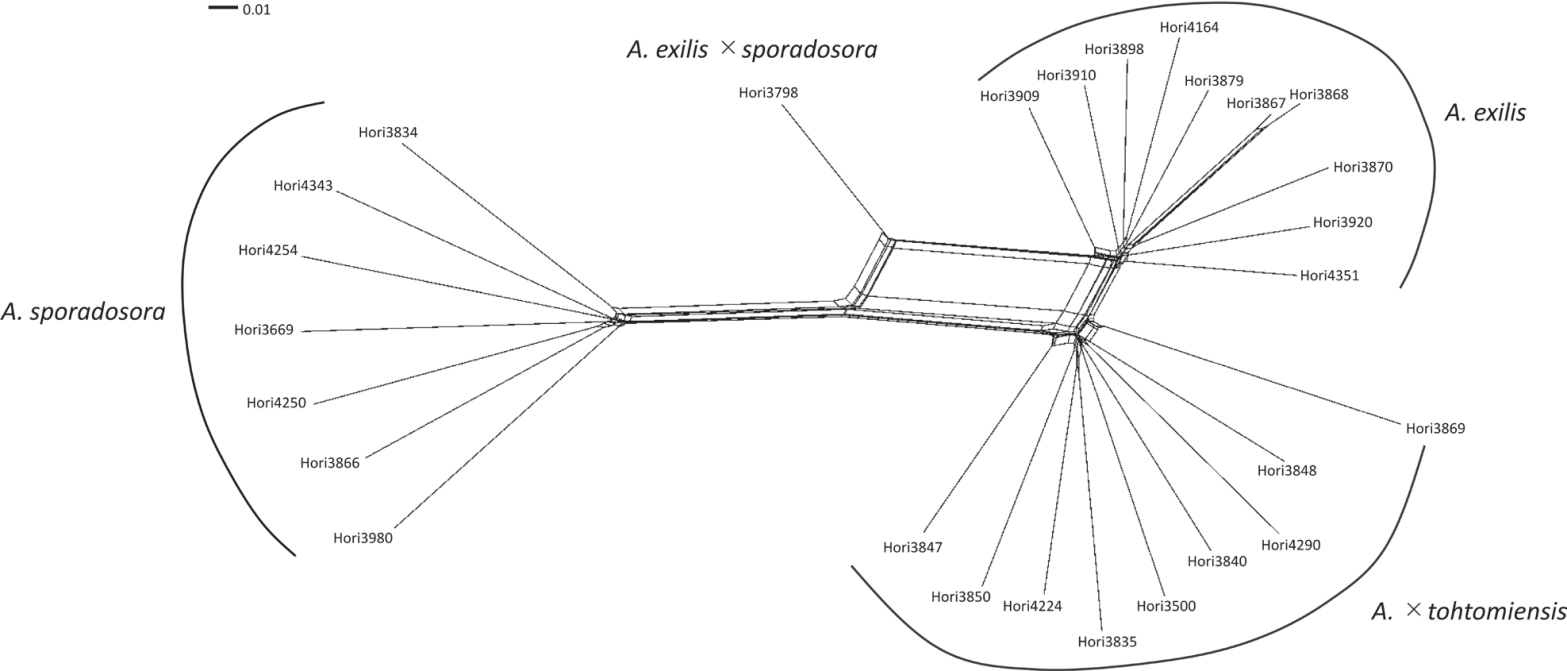
Neighbor-Net network of *Arachniodes × tohtomiensis* and its relatives using SNP data from MIG-seq.

In the neighbor-net network (Fig. 5), *Arachniodes exilis*, *A. sporadosora*, and *A. × tohtomiensis* formed three separate clusters, whereas *Arachniodes exilis* × *A. sporadosora* occupied an intermediate position, with reticulations connecting its parental species. *A. exilis* and *A. × tohtomiensis* formed two neighboring clusters.

The morphological characteristics of *Arachniodes exilis*, *A. sporadosora*, *A. exilis* × *A. sporadosora*, and *A. × tohtomiensis* are summarized in Table 1. *A. × tohtomiensis* is characterized by yellowish-green, slightly glossy laminae, and erose or ciliate indusia. *A. exilis*, *A. exilis* × *A. sporadosora*, and *A. × tohtomiensis* form dense forest floor populations with long creeping rhizomes (>15 cm), whereas *A. sporadosora* grows scattered with short rhizomes (<15 cm). The lamina color is yellowish-green and slightly glossy in *A. × tohtomiensis* in contrast to dark green and glossy in the others. The lamina texture is papyraceous in *A. exilis* and *A. × tohtomiensis*, coriaceous or papyraceous in *A. exilis* × *A. sporadosora*, and coriaceous in *A. sporadosora*. Lamina dissection varies as follows: tripinnate pinnatifid to tripinnate in *A. sporadosora* and *A. × tohtomiensis*, bi- to tripinnate pinnatifid in *A. exilis* × *A. sporadosora*, and bi- to tripinnate in *A. exilis*. The lamina apex subabruptly narrows in *A. exilis* and *A. × tohtomiensis*, gradually or subabruptly narrows in *A. exilis* × *A. sporadosora*, and gradually narrows in *A. sporadosora*. The stipe spacing on the rhizome is 4–5 cm in *A. exilis* and *A. × tohtomiensis*, 2–4 cm in *A. exilis* × *A. sporadosora*, and <1.5 cm in *A. sporadosora*. The indusia are erose or ciliate in *A. × tohtomiensis*, whereas entire in *A. exilis* and *A. exilis* × *A. sporadosora*, and usually entire (rarely slightly erose) in *A. sporadosora*.

**Table 1.** Morphological characteristics of *A. exilis*, *A. sporadosora*, *A. exilis* ×*A. sporadosora*, and *A. × tohtomiensis*.

Characteristics/Species	* A. exilis*	* A. sporadosora*	* A. exilis* × *A. sporadosora*	* A. × tohtomiensis*
Rhizomes	longer than 15 cm	shorter than 15 cm	longer than 15 cm	longer than 15 cm
Stipe spacing on rhizomes	4–5 cm	less than 1.5 cm	2–4 cm	4–5 cm
Color of adaxial surface of laminae	glossy dark-green	glossy dark-green	glossy dark-green	slightly glossy yellowish-green
Dissection at middle of laminae	bipinnate to tripinnate	tripinnate pinnatifid to tripinnate	bipinnate to tripinnate pinnatifid	tripinnate pinnatifid to tripinnate
Texture of laminae	papyraceous	coriaceous	coriaceous or papyraceous	papyraceous
Apex of laminae	narrow subabruptly	narrow gradually	narrow gradually or subabruptly	narrow subabruptly
Margin of indusia	entire	usually entire (rarelly erose)	entire	erose or ciliate

## Discussion

Plastid DNA phylogeny, STRUCTURE analysis, neighbor-net, and PCA consistently indicated that *Arachniodes × tohtomiensis* is a hybrid origin of *A. exilis* and undetected species. The plastid DNA phylogeny suggested that either *A. exilis* or an undetected parent was the maternal parent because seven samples had haplotype A (undetected species), and two samples had haplotype B (*A. exilis*). Evidence of an unknown genome was also provided through STRUCTURE analysis and PCA. No individuals were discovered that represented the pure genome of the undetected species.

*Arachniodes exilis* and *A. sporadosora* contributed to the formation of *A. exilis* × *A. sporadosora*, which is consistent with the morphological evidence and findings of previous molecular studies (Tsutsui 1988; Hori 2024). The maternal parent of the material analyzed in this study was likely *A. sporadosora*, which possesses the plastid haplotype C2. F1 hybrids of *A. exilis* × *A. sporadosora* produce viable spores capable of forming F2 hybrids with *A. exilis*; thus, recognizing this hybrid is challenging because of the continuous morphological variation between the parental species (Shimura et al. 1982; Tsutsui 1988). In contrast, *A. × tohtomiensis* contrastingly exhibited a relatively distinctive morphology, with slightly glossy yellowish-green laminae, and indusia erose or ciliate on margins. A hybrid may be formally described if at least one parent is known according to the International Code of Nomenclature for Algae, Fungi, and Plants (Turland et al. 2025). Although no known species matched the morphology inferred for this undetected parent based on the descriptions of Sino-Japanese species (He et al. 2013), the formal description of *A. × tohtomiensis* in this study represents an important step toward clarifying the taxonomic and phylogenetic diversity of the genus *Arachniodes* in the Sino-Japanese region.

The phylogenetic tree based on plastid DNA sequences (Fig. 1) did not support the taxonomic treatment of Chang et al. (2021), who synonymized *Arachniodes exilis* with *A. carvifolia* and *A. sporadosora* with *A. cornu-cervi*. Instead, it showed that *A. exilis* and *A. sporadosora* found in Japan are not closely related to these Chinese species; *A. sporadosora* is closely related to *A. carvifolia*, but distinct from it. Therefore, the treatment of these taxa as distinct species (Ebihara 2017; Lu et al. 2019) is supported.

The DNA content indicates that the materials examined in this study were diploid. The reported DNA contents are comparable to those of known diploid species and approximately half of those of tetraploid species in *Arachniodes* (Hori and Ichihara 2024; Hori et al. 2025). Therefore, *A. × tohtomiensis* is not an allopolyploid species, and polyploidization is not seemed to be occurred.

## Taxonomic treatment

### ﻿ Arachniodes × tohtomiensis

Taxon classificationPlantaePolypodialesDryopteridaceae

Shimura & Hori, hyb. nov.

544E6694-46DE-579E-94A8-41ECEB237824

urn:lsid:ipni.org:names:77375094-1

Figs 6, 7, 8

— Arachniodes × tohtomiensis Shimura, Enum. Vasc. Pl. Fukuoka Pref. Jap. 1. Pterid.: 90. 1988, nom. nud.

#### Note.

An interspecific hybrid between *A. exilis* (Hance) Ching and an unknown species.

**Figure 6.**
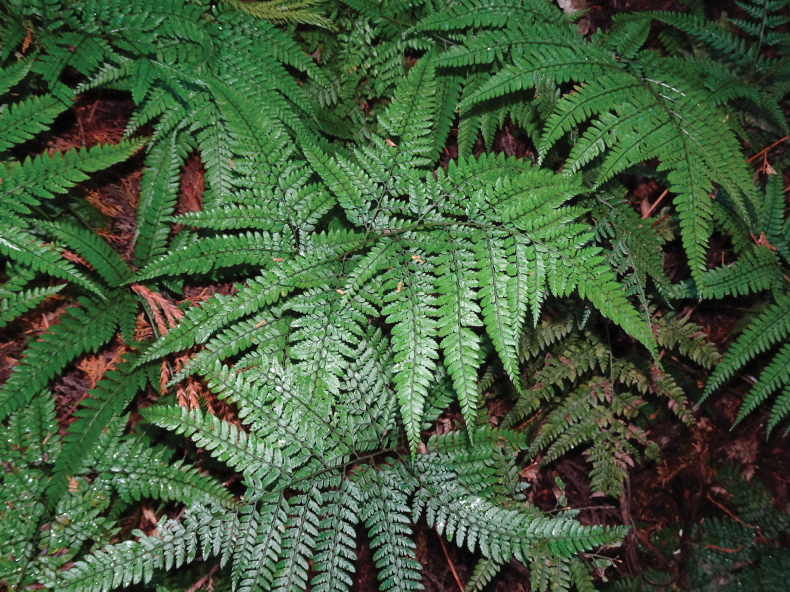
*
Arachniodes × tohtomiensis* in its natural habitat.

**Figure 7.** *
Arachniodes × tohtomiensis* Shimura & Hori. **A**. Habit; **B**. Secondary pinnule; **C**. Lower stipe scale; **D**. Scale on abaxially surface of secondary pinnule; **E**. Indusium. **A–E**. From the holotype (*Hori 3840*, MBK0340006) (illustration by K. Hori).

**Figure 8.**
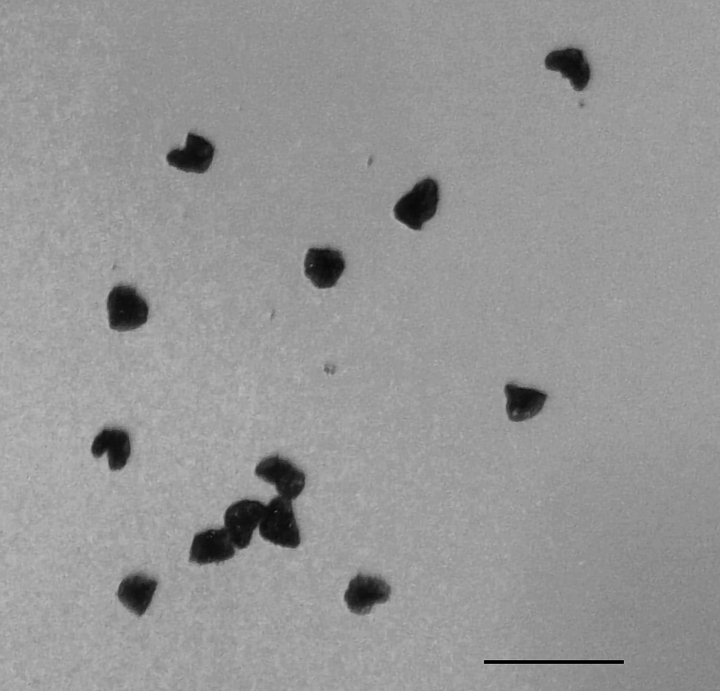
Irregular-shaped shrunken spores of *Arachniodes × tohtomiensis*. Scale bar: 100 μm.

#### Diagnosis.

*Arachniodes × tohtomiensis* is similar to *A. exilis*, *A. exilis* × *A. sporadosora*, and *A. sporadosora* in their aristate secondary pinnules. However, *A. exilis*, *A. exilis* × *A. sporadosora*, and *A. sporadosora* have glossy dark-green laminae adaxially and indusia entire on margins. The rhizomes of *A. sporadosora* are shorter than 15 cm. In contrast, *A. × tohtomiensis* has slightly glossy yellowish-green laminae, indusia erose or ciliate on margins, and rhizomes longer than 15 cm.

#### Type.

Japan. Honshu: Kyoto-fu, Fukuchiyama city, Tano, 35°13'38.56"N, 135°09'47.64"E, alt. 158 m, coniferous forest of *Cryptomeria japonica* (L.f.) D.Don, on soil, 8 Jan 2023, *K. Hori 3840* (holotype: MBK0340006, isotype: MBK0340007, 0340008).

#### Description.

***Terrestrial evergreen fern*. *Rhizomes***: long creeping, occasionally branched, often forming pure forest population, 15–40 cm × 1–3 cm, sparsely set with roots and persistent, densely clothed with reddish-brown scales; ***fronds***: 2–5 per rhizome; ***stipes***: stramineous, spacing on the rhizome 4–5 cm, 15–40 cm × 0.2–0.3 cm, clothed with bullate black scales (2.2–7.5 mm × 0.5–1.0 mm, entire on margin) densely in basal, rather sparsely in middle to upper sections; ***laminae***: slightly glossy yellowish-green on adaxial surface, quadripinnate pinnatifid at base, tripinnate pinnatifid to tripinnate at middle, bipinnate at apex, 35–40 cm × 25–37 cm, ovate-pentagonal or deltoid-pentagonal, papyraceous, apex subabruptly narrowed and elongated acute; ***rachises***: stramineous, rather densely scaly; ***pinnae***: 4–7 pairs, obliquely spreading, alternate, petiolated (2.5–6.0 mm long), lowest pair largest, lanceolate, 15–17 cm × 15–17 cm; ***primary pinnules***: truncate toward the base, asymmetrical, acute at apex, 20–25 pairs at middle part of laminae, 1–20 pairs at upper part of laminae, basal basiscopic one or two and acroscopic one elongated (8–13 cm × 1.5–2.8 cm and shortly stalked), distally abruptly shortened and subsessile; ***secondary pinnules***: deltoid-oblong, rather symmetrical, attenuate toward the base, obtuse at apex, ca. 0.6–1.8 cm × 0.3–1.2 cm, margin serrate to deeply pinnatifid, aristate, axes and veins covered with minute brown linear-subulate scales abaxially; ***the most basiscopic pinnules on the lowest pinnae***: lanceolate, abruptly elongated than second basiscopic pinnules on the lowest pinnae, petiolated (1–3 mm long), 10–13 cm × 1.8–2.5 cm; ***sori***: terminal on veinlets, 1–5 pairs per primary or secondary pinnules, medial between midvein and margin; ***indusia***: cloudy white, reniform, erose or ciliate on margin, persistent; ***spores***: irregular shaped, abortive, without ability of growing.

#### Etymology.

The name derives from ‘tohtomi’ which mean Shizuoka prefecture in Japanese where *Arachniodes × tohtomiensis* was initially found.

#### Additional specimens examined.

**Japan**. • **Honshu**: Shimane prefecture, Yasugi city, Hirose-cho, alt. 82 m, coniferous forest of *C. japonica*, on soil slope, 24 Aug 2024, *K. Hori 4290* (MBK0351459). Shizuoka prefecture, Iwata city, Kaminobe, alt. 49 m, coniferous forest of *C. japonica*, on soil, 21 Jan 2023, *K. Hori 3869* (MBK0340038). ibid., Jul 6 1980, *Y. Shimura s.n*. (TNS-VS-537144, diploid sterile, photo!). • **Shikoku**: Ehime prefecture, Saijo city, cultivated at the Kochi Prefectural Makino Botanical Garden, 19 Mar 2021, *K. Hori 3500* (MBK0329364). Kochi prefecture, cultivated at the Kochi Prefectural Makino Botanical Garden, 4 Jan 2023, *K. Hori 3835* (MBK0339966). • **Kyushu**: Fukuoka prefecture, Fukuoka city, Mt. Katanawa, alt. 191 m, coniferous forest of *C. japonica*, on soil, 9 Jan 2023, *K. Hori 3847* (MBK0340015). ibid., Miyawaka-city, Rikimaru, alt. 49 m, on soil, 9 Jan 2023, *K. Hori 3848* (MBK0340016). ibid., Kasuya-gun, Todoroki, alt. 191 m, coniferous forest of *C. japonica*, on soil, 9 Jan 2023, *K. Hori 3850* (MBK0340018). Kumamoto prefecture, Amakusa city, Amakusa machi, Takahamakita, 7 April 2024, *K. Hori 4224* (MBK0353331).

#### Distribution and ecology.

This hybrid is a naturally occurring taxon restricted to western Japan, growing on soil under coniferous or evergreen forests.

## Supplementary Material

XML Treatment for Arachniodes × tohtomiensis

## Additional information

### Use of AI

No use of AI was reported.

### Author ORCIDs

Kiyotaka Hori https://orcid.org/0000-0002-6815-7595

Daiki Takahashi https://orcid.org/0000-0002-4105-5417

Yoshihisa Suyama https://orcid.org/0000-0002-3136-5489

### Data availability

All of the data that support the findings of this study are available in the main text or Supplementary Information.

## Supplementary materials

Supplementary material 1 List of the samples in this study Data type xlsx https://binary.pensoft.net/file/1513547This dataset is made available under the Open Database License (http://opendatacommons.org/licenses/odbl/1.0/). The Open Database License (ODbL) is a license agreement intended to allow users to freely share, modify, and use this Dataset while maintaining this same freedom for others, provided that the original source and author(s) are credited.Author: Kiyotaka Hori
